# Intercellular Communication of Tumor Cells and Immune Cells after Exposure to Different Ionizing Radiation Qualities

**DOI:** 10.3389/fimmu.2017.00664

**Published:** 2017-06-07

**Authors:** Sebastian Diegeler, Christine E. Hellweg

**Affiliations:** ^1^Division of Radiation Biology, Institute of Aerospace Medicine, German Aerospace Center (DLR), Köln, Germany

**Keywords:** radiation-induced bystander effects, natural killer cells, cytotoxic T-cells, cytokines, radiotherapy

## Abstract

Ionizing radiation can affect the immune system in many ways. Depending on the situation, the whole body or parts of the body can be acutely or chronically exposed to different radiation qualities. In tumor radiotherapy, a fractionated exposure of the tumor (and surrounding tissues) is applied to kill the tumor cells. Currently, mostly photons, and also electrons, neutrons, protons, and heavier particles such as carbon ions, are used in radiotherapy. Tumor elimination can be supported by an effective immune response. In recent years, much progress has been achieved in the understanding of basic interactions between the irradiated tumor and the immune system. Here, direct and indirect effects of radiation on immune cells have to be considered. Lymphocytes for example are known to be highly radiosensitive. One important factor in indirect interactions is the radiation-induced bystander effect which can be initiated in unexposed cells by expression of cytokines of the irradiated cells and by direct exchange of molecules *via* gap junctions. In this review, we summarize the current knowledge about the indirect effects observed after exposure to different radiation qualities. The different immune cell populations important for the tumor immune response are natural killer cells, dendritic cells, and CD8+ cytotoxic T-cells. *In vitro* and *in vivo* studies have revealed the modulation of their functions due to ionizing radiation exposure of tumor cells. After radiation exposure, cytokines are produced by exposed tumor and immune cells and a modulated expression profile has also been observed in bystander immune cells. Release of damage-associated molecular patterns by irradiated tumor cells is another factor in immune activation. In conclusion, both immune-activating and -suppressing effects can occur. Enhancing or inhibiting these effects, respectively, could contribute to modified tumor cell killing after radiotherapy.

## Introduction

**Figure 1:**
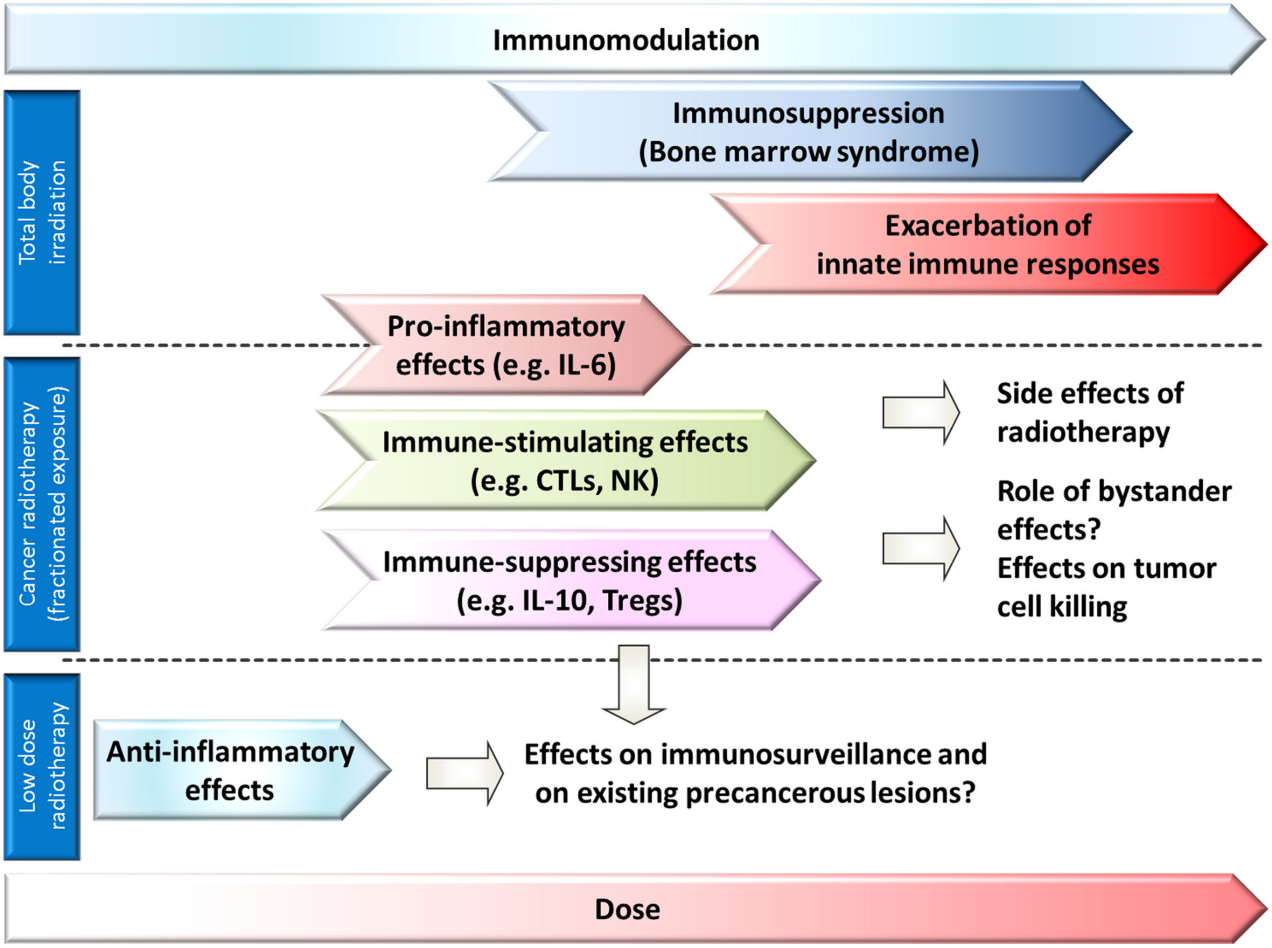
Role of immune responses and affection of the immune system in different dose ranges after whole-body exposure (or bone marrow exposure) or partial body exposure. A modulation of immune responses can be expected in all dose ranges. Anti-inflammatory effects are observed in low-dose radiotherapy (partial body exposure), and proinflammatory and immune stimulating effects in some tumor radiotherapy settings (partial body exposure), but also immune-suppressing effects might occur. In whole-body exposure to medium to high doses of ionizing radiation, exacerbation of innate immune responses, and bone marrow depression dominate the picture of acute radiation sickness.

In the response to radiation exposure, interactions with the immune system play an important role at multiple levels. Different exposure conditions [e.g., partial body/total body, dose and dose rate, fractionation, acute or chronic, radiation quality as determined by linear energy transfer (LET)] are expected to modulate the immune system in many ways. A concept of the complex involvement of the immune system in the organismal response to whole-body or partial body irradiation is suggested in Figure 1.

First of all, immune cells and their lymphoid and myeloid precursors and stem cells can be affected directly. These effects are of major importance for acute medium- to high-dose exposures to ionizing radiation as the hematopoietic system. Self-renewing hematopoietic stem cells (HSCs) and more differentiated hematopoietic progenitor cells (HPCs) in the bone marrow are extremely radiosensitive (1) because of their rapid turnover. Also, some of the mature cells from the different lineages such as lymphocytes are sensitive to ionizing radiation. Depletion of already differentiated cells by cell death mechanisms and failing replacement by stem cells due to cell death [apoptosis of HPCs and HSCs (2)] or increased p21^Cip1/Waf1^ (Gene name: *Cdkn1a*, cyclin dependent kinase inhibitor 1 A) expression leading to a cell cycle block and loss of clonogenic function (2) severely affects the immune system. Only cells overcoming the cell cycle block are able to replace radiation-damaged tissue to regain normal function.

Therefore, immunodepression is a predominant feature of acute radiation sickness (bone marrow or hematopoietic syndrome) and it appears after whole-body exposure to doses of 0.5–4 Gy (Figure 1) (3, 4). In the bone marrow syndrome, progressive lymphopenia develops during the first days after radiation exposure. Exposure to ~2 Gy results in maximal depression of the lymphocytes in the blood (5). The lymphocyte deprivation decreases the resistance to infections. A possible early granulocytosis is followed by a progressive granulocytopenia (6). Death usually occurs from sepsis at 30–60 days after radiation exposure, if the patient cannot be carried through the critical period of the possibly reversible aplastic state of the bone marrow (5). Long-term persisting damage (up to 16 months in mice) of HSCs is observed after a single acute high-dose exposure (7). Cytological abnormalities (multipolar mitosis, micronuclei, mitotic bridges, and binucleated cells) and a reduced mitotic index were observed in human bone marrow cells (e.g., erythroblasts) during the first days after accidental sublethal whole-body γ-radiation exposure, and they persist at a lower frequency for years after the accident (8). The long-term bone marrow injury after acute exposure to moderate of high doses of low-LET-irradiation might be caused by HSC senescence (9) as indicated by increased p16^Ink4a^ expression and senescence-associated-β-galactosidase activity (2). Radiation qualities with higher biological effectiveness such as accelerated iron ions, exhibiting a different LET depending on charge and energy of the ion, were shown to initiate long-term damage to hematopoietic early and late multipotent progenitor cells in mice and reprogramming to a primitive pluripotent state (1). Furthermore, chronic low-dose exposure to ionizing radiation might damage bone marrow cells as the hematopoietic niche is regarded to be highly sensitive to low-dose ionizing radiation exposure (Figure 1) (1).

In addition to the well-known immunosuppression as the predominant feature of the bone marrow syndrome, recent studies suggest that in the acute radiation syndrome, exacerbated innate immune responses play a major role in pathogenesis (10–12). Epithelial and endothelial cells are suggested as source of the proinflammatory cytokines in the acute radiation syndrome (12). In this complex chain of events, endothelial cells and parenchymal cells are damaged (13), endothelial cells and leukocytes are activated, proinflammatory cytokines such as interleukin-8 (IL-8), IL-6, IL-12 and IL-18, prostaglandin E2 (PGE2) and reactive oxygen species (ROS) are produced (10, 14), and neuropeptides are released (15). Activation of the innate immune system was suggested to be involved in target organ damage and adverse metabolic and hemodynamic responses (10). In the brain, overexpression of cytokines such as tumor necrosis factor α (TNF-α), IL-1α, and IL-1β occurs within several hours after whole-body irradiation of mice (10).

Partial body irradiation is applied in tumor radiotherapy or can occur in radiation accidents. Short-term side effects of conventional radiotherapy depend on the location, the total dose of radiation treatment, the individual radiosensitivity, and the size of the radiation field. A persistent accumulation and activation of immune cells (e.g., macrophages), resulting in the release of proinflammatory cytokines (IL-1, IL-6), contributes to radiotherapy-induced side effects (10) such as cutaneous radiation syndrome, oral mucositis, radiation pneumonitis or esophagitis, or cystitis (16–18). Furthermore, the cytokine transforming growth factor β (TGF-β) might be activated in the extracellular space and upregulation of its receptors might deregulate fibroblast proliferation and differentiation and contribute to radiation-induced fibrosis (19).

Accelerated ion species, especially protons and carbon ions, are already established features of state-of-the-art radiotherapy. One of their main physical properties is a distance-controlled energy distribution (Bragg Peak), resulting in highly localized energy deposition of radiation with high LET within a tumor while at the same time protecting out-of-field tissue from exposure due to low entry- and even less exit-energies. Such level of radiation control makes this therapeutic approach especially suitable for treatment in unfavorable locations and strongly promotes personalized therapy.

The direct effects of ionizing radiation exposure on different immune cells and their stem cells and especially their radiation sensitivity were recently summarized in three reviews (20–22), therefore, the readers are referred to these reviews and other reviews for a detailed description of the immune cells, an overview of their function and the direct radiation effects. Shortly, granulocytes (eosinophils, basophils, neutrophils), natural killer (NK) cells and mast cells are the major players in the innate immune system. T-lymphocytes with their subtypes [cytotoxic T-cells (CTLs), helper T-cells (Ths) with the subpopulations Th1, Th2, Th17, regulatory T-cells (Tregs), memory T-cells] and B-lymphocytes (23) [plasma cells, and memory B cells] represent the adaptive arm of the immune system. T-lymphocytes are the key players in the cell-mediated immune response, while B-lymphocytes mediate the humoral reactions. The circulating peripheral blood lymphocytes represent only <2% of the lymphocytes in lymphoid tissues (24). At the interface of the innate and the adaptive immune system, macrophages derived from monocytes and dendritic cells (DCs) act as antigen-presenting cells (APCs). NKT cells show features of NK cells and T-lymphocytes. The direct effects encompass reduced survival, proliferation, cell cycle alterations, diminished function, gene expression changes (25–27), chromosomal aberrations, mutations, and possible transformation (28). *In vivo*, mitotic catastrophe is usually followed by necrosis resulting in an inflammatory reaction (29, 30). Mitotic catastrophe contributes strongly to the death of tumor cells induced by ionizing radiation (29), and is now assumed to be the major cell death pathway in solid tumors following radiotherapy (31). In tumor radiotherapy, this might result in enhanced tumor cell killing by cytotoxic immune cells and also in damage to the normal tissue (32).

More subtle changes are expected at low doses, and the bystander effect as a non-targeted effect being expressed in unexposed cells which are in the vicinity of irradiated cells, becomes apparent when only a small fraction of cells was hit. Such bystander effects are also relevant in radiotherapy dose ranges, as immune cells can enter the irradiated tumor tissue and interact with the irradiated tumor cells. They are of high importance for cancer immunotherapy concepts in combination with radiotherapy in which unirradiated immune cells are to be injected in the tumor/the patient. Also, the effects on immune cells in their niche—mesenchymal stem cells (33, 34) and endothelial cells (35) are in the focus of current research activities. Furthermore, abscopal effects, which are observed in non-irradiated fields after localized radiation exposure, have been recognized for decades, most particularly after radiotherapy (12).

In this review, we discuss the intercellular communication in the tumor immune response with a focus on different ionizing radiation qualities. This encompasses the recruitment of immune cells to the irradiation site by, e.g., chemokines, and the functional modulation of immune cells.

## Radiation-Induced Bystander Effects

Ionizing radiation, whether it is photonic radiation like X-rays and γ-rays or accelerated high energy particles, affects not only the cells they are exposed to. Radiation-induced bystander effects (RIBEs) are a response of cells that are not directly hit by ionizing photons or traversed by heavy ion species that is initiated by cells which received doses of ionizing radiation (36).

After an ionizing radiation event damages a cell, pathways leading to the repair of the damages or the induction of apoptosis also induce the production of factors that can travel outside of the cell or from cell to cell, either by secretion or *via* cell-to-cell connecting channels. These factors act as damaging agents or signaling molecules and can affect other cells in a paracrine or endocrine manner.

Radiation-induced bystander effects have been first described by Nagasawa and Little in an experiment, where only a small fraction of the cells (<1%) were traversed by an α-particle, but more than 30% of the whole cell population showed damages (37). At present time, damages by RIBE are characterized as DNA damage, chromosome aberrations, sister-chromatid exchanges, genomic instability, and cellular senescence. Among the damaging agents are ROS and reactive nitrogen species (RNS) (38, 39).

Radiation-induced bystander effects are not only an indirect way for ionizing radiation to cause destruction. The secretion of signaling factors of this particular cellular response can also protect cells from further damages by preenhancing repair mechanisms or lead to a faster clean-up of radiation-damaged cells (40–42).

The most prominent signaling molecules in RIBE are factors triggering an immune response. Part of the damage response of an irradiated cell is the activation of the transcription factor nuclear factor κB (NF-κB) (43). Downstream of NF-κB activation, chemokines and cytokines are produced and secreted, which can attract and stimulate cells of the immune system.

Besides cytokine and chemokine secretion, cells can communicate *via* extracellular vesicles or exosomes. These membrane-coated bodies can contain a multitude of factors ranging from proteins to micro-RNA that can modulate cellular functions and induce signaling pathways. After secretion of the vesicles into the extracellular space, exosomes can affect neighboring cells by binding to surface receptors or by uptake and intracellular release of their content. Exosomes in RIBE have been associated with DNA damage, survival, proliferation, and signal transduction, resulting from the variety of factors carried within and the possible ways to impact recipient cells (44–52). The influence of ionizing radiation on composition and secretion of exosomes was recently reviewed by Jelonek et al. (49).

In the innate immune response, recognition of pathogen-associated molecular patterns or damage-associated molecular patterns (DAMPs) by germline-coded cell surface or intracellular receptors [pattern recognition receptors (PRRs)] is the central trigger of activation. In the adaptive immune response, antigen presentation by APCs to T- and B-lymphocytes is the central process for their activation. Antigens are bound to major histocompatibility complex class I (MHC-I) molecules on the surface of body cells and to MHC class II (MHC-II) molecules on APCs [in humans: MHC class Ia – human leukocyte antigen (HLA)-A, -B and -C; MHC class Ib – HLA-E, -F-, -G; MHC class II – HLA-DM, -DO, -DP, -DQ, -DR]. Antigen recognition by T helper cells and B-cells or CTL in combination with co-stimulation, intercellular adhesion and stimulation by cytokines results in their activation. Therefore, radiation induced modifications of these intercellular communication pathways are of utmost importance in the non-targeted response of the immune system.

Radiation-induced bystander effects in the immune system encompass a complex network of signaling pathways, ranging from the DNA damage response of irradiated cells and unirradiated cells over the regulation of surface molecules on stationary body cells as well as circulating immune cells after radiation exposure and on the non-irradiated neighbors to the response of immune cells, due to direct or indirect intercellular communications of immune cell populations.

*In vitro* experiments for analysis of RIBE are based on transfer of conditioned medium from irradiated cells on unirradiated cells, coculture of irradiated and unirradiated cells, or irradiation of a subpopulation of cells by means of a microbeam or partial shielding.

## Action of Immune Cells after Tumor Irradiation

Tumors contain diverse immune cells, and therefore, the responses of immune cells to irradiated tumor cells including RIBE are an important factor for the overall outcome of the tumor therapy. Noteworthy for this topic is the strict differentiation of *in vitro* and *in vivo* studies: *in vitro* experiments with unirradiated immune cells can show an uncompromised immune response against irradiated tumor cells. In *in vivo* studies, immune cells may also be irradiated during radiation therapy of the experimental tumor.

The responses of immune cells to stresses of any kind differ as much as their population diversity. While there are actively lytic cell populations, such as CD8+ CTLs and NK cells, there is also a host of immune actions that are necessary for initiating aforementioned lytic responses (e.g., dendritic, monocytic, and macrophage-mediated presentation of antigens) and enhancing actions (Th1 and Th2 responses). Opposed to those proinflammatory lymphocytes are cell populations that suppress the responses, for example, Tregs that secrete the hematopoietic cell activity regulating and anti-inflammatory TGF-β and the immune-suppressing IL-10 (53).

### Activation of CTLs

**Figure 2:**
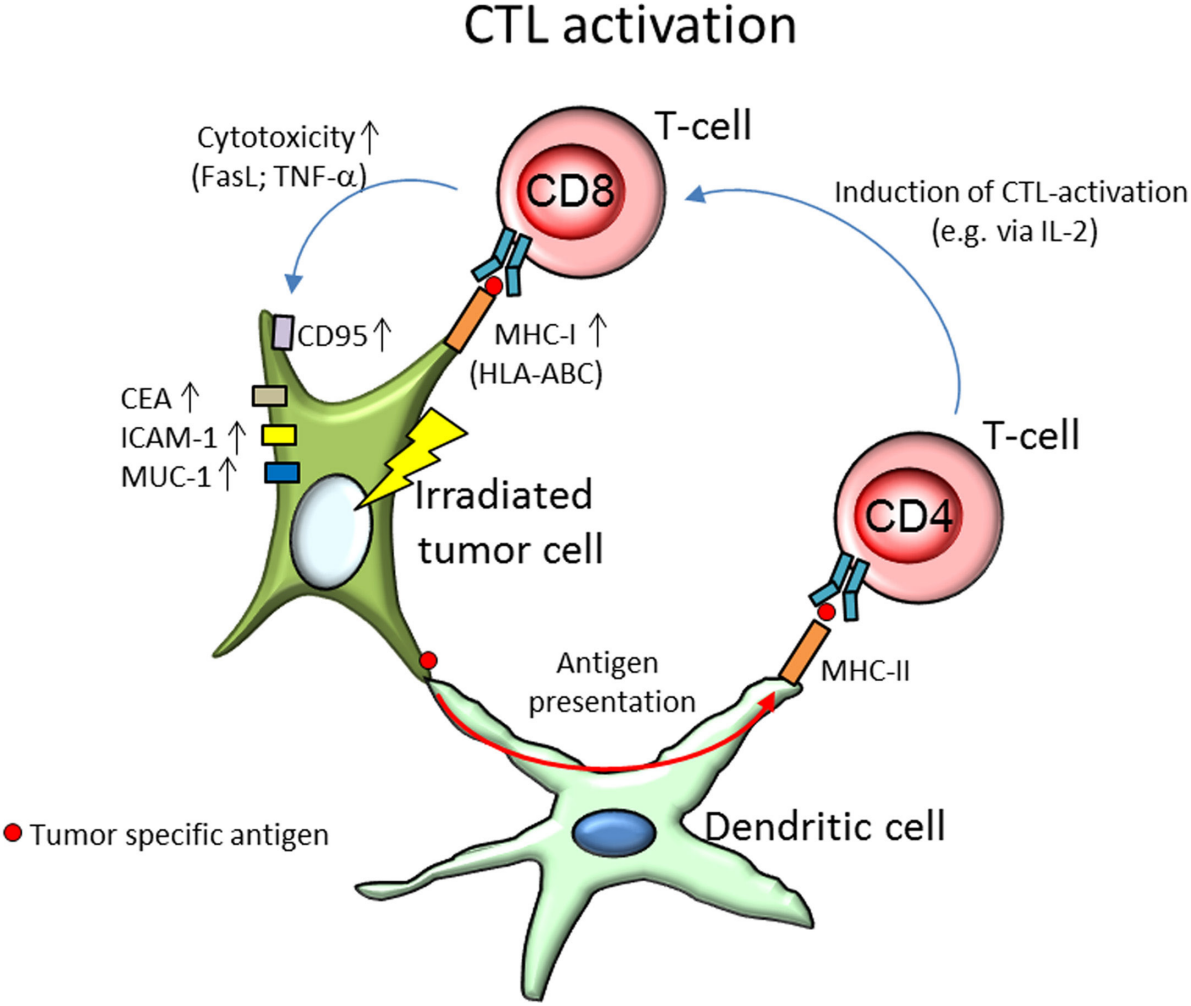
Activation of CD8+ cytotoxic T-cells (CTL) by tumor specific antigens presented by the irradiated tumor cell and dendritic cells (DCs). After irradiation, the tumor cell shows an increased expression of surface markers CD95 (Fas), carcinoembryonic antigen (CEA), intercellular adhesion molecule 1 (ICAM-1), and mucin-1 (MUC-1), as well as upregulated expression of major histocompatibility complex class I (MHC-I; HLA-ABC, human leukocyte antigen A, B, and C). While increased expression of CEA, ICAM-1, and MUC-1 are found to enhance cytolytic T-cell activity, CD95, and MHC-I are responsible for the activation of the T-cell. Increased expression of either has been associated with elevated activation of CTL. By binding with surface bound Fas-ligand (FasL) to the tumors’ CD95, T-cells can initiate tumor cell death *via* apoptosis. MHC-I molecules on the other hand present tumor specific antigens to the T-cell *via* the T-cell receptor and initiate degranulation of tumor necrosis factor α (TNF-α), perforines, and granzymes, thereby lysing the target tumor cell. After irradiation, tumor cells were found to produce unique antigen peptides, leading to increased tumor recognition. DCs, in their role as antigen-presenting cells, enable radiation-induced CTL lysis. DC take up tumor specific antigens and present them *via* MHC-II molecules to T-helper cells (CD4+), which prime and activate CTL, e.g., *via* secretion of interleukin-2 (IL-2).

Involvement of cytotoxic immune cells has been studied in a variety of model systems with different radiation qualities. The most notable modifications of lymphocyte actions are summarized in Table 1. Activation of CTL (shown in Figure 2) is mainly triggered *via* the T-cell receptor. In 67NR and A20 tumor-bearing mice irradiated with γ-rays (2–6 Gy), increased CTL cytotoxicity was reported (54). In an *in vitro* study by Garnett et al. (55) using several carcinoma cell lines, it was shown that after irradiation with γ-rays (10–20 Gy), WiDr, Caco-2, SW620, SW1463, and HCT116 cells were more sensitive to CTL-mediated lysis primed against carcinoembryonic antigen (CEA), while A549 cells responded to Fas-mediated cell lysis. Increased expression of Fas (CD95) was also observed on tumor cells in a MC38 mouse adenocarcinoma cell model after γ-irradiation (20 Gy), which enhanced the lytic activity of CTL (56). Expression of the surface proteins Fas, CEA, intercellular adhesion molecule 1 (ICAM-1), mucin-1 (MUC-1), and MHC-1 was increased in those cell lines as well, enhancing their susceptibility to immune mediated lysis (55). ICAM-1 can engage in receptor-ligand binding between a T-cell and an antigen-presenting DC and thereby contribute to T-cell activation (21) as well as recruitment of immune cells from the blood stream to endothelial cells before extravasation to the tumor (57, 58).

**Table 1:** Modulation of lymphocyte activity after irradiation of tumor tissue.

Tumor cell	Radiation quality	Dose	Study type	Lymphocyte type	Activity	Reference
Mouse adenocarcinoma	γ-Irradiation (^137^Cs source)	20 Gy	*In vivo*	CTL	⇑	(56)

67NR (breast)	γ-Irradiation (^60^Co source)	2–6 Gy	*In vivo*	CTL	⇑	(54)
A20 (lymphoma)						

WiDr (colon)	γ-Irradiation (^137^Cs source)	10–20 Gy	*In vitro*	CTL	⇑	(55)
Caco-2 (colon)						
SW620 (colon)						
SW1463 (colon)						
HCT116 (colon)						
A549						

MelJuSo (melanoma)	γ-Irradiation (^137^Cs source)	1–30 Gy	*In vitro*	CTL	⇑	(59)

RMA-S lymphoma	*Radiation therapy (presumed X-rays)*	^a^	*In vivo*	NK	⇑	(60)
B16 melanoma						

A549 (lung carcinoma)	X-rays exposure (ClinaciX Linear Accelerator)	8 Gy	*In vitro*	NK	⇑	(61)
NCI-H23 (lung adenocarcinoma)						

MDA-MB-231 (breast)	Electron beam exposure (Elekta Synergy linear accelerator)	8 Gy	*In vivo*	NK	⇑	(62)
U87MG (glioblastoma)						
A673 (muscle)						
PANC-1 (pancreas)						

Lewis Lung carcinoma	X-rays exposure (6-MV photon beam, dose rate 6.1 Gy/min)	12 Gy	*In vivo*	Treg	⇑	(63)
CT-26 colon carcinoma						

B16 melanoma	γ-Irradiation (^137^Cs source)	6–12 Gy	*In vivo*	Treg	⇑	(64)
EL-4 lymphoma						

PANC-02 (pancreas)	γ-Irradiation (Siemens Gammatron)	5 Gy × 2 Gy	*In vivo*	CTL, NK	CTL > NK	(65)

LNCaP (prostate)	γ-Irradiation (^137^Cs source)	8 Gy	*In vivo*	CTL	⇑	(66)
MDA-MB-231 (breast)						
H1703 (lung)	Proton ion irradiation (200 MeV, LET 0.5 keV/µm)	8 Gy				
JHC7 (chordoma)						

Mouse SCCVII (squamous cell carcinoma)	Carbon ion irradiation (290 MeV/n, LET 77 keV/µm)	10 Gy/min^b^	*In vivo*	CTL+ DC	⇑	(67)

*⇑ up*.

*^a^Dose not indicated*.

*^b^Duration of irradiation not indicated*.

*LET, linear energy transfer*.

Similar results were obtained using 200 MeV protons (produced using a passive scattering proton beam). In *in vitro* tumor cell models (human prostate (LNCaP), breast (MDA-MB-231), lung (H1703) carcinoma, and chordoma (JHC7) cells), expression of HLA-ABC, CEA, MUC-1, and ICAM-1 was increased after proton (8 Gy) and γ-irradiation (8 Gy), as well as sensitivity of the tumor cells to CEA-specific CTL-mediated lysis increased (66). Increased CTL activity has been partially allotted to the production of unique MHC-I antigenic peptides after γ-irradiation (1–25 Gy) leading to increased tumor recognition by T-cells (59).

*In vivo* studies with carbon ion irradiation (290 MeV/n, LET 77 keV/µm) of tumor-bearing mice revealed an increased CTL-associated lysis of isolated tumor splenocytes after carbon ion irradiation treatment with supplementary intratumoral DC injection (67).

### Activation of NK Cells

**Figure 3:**
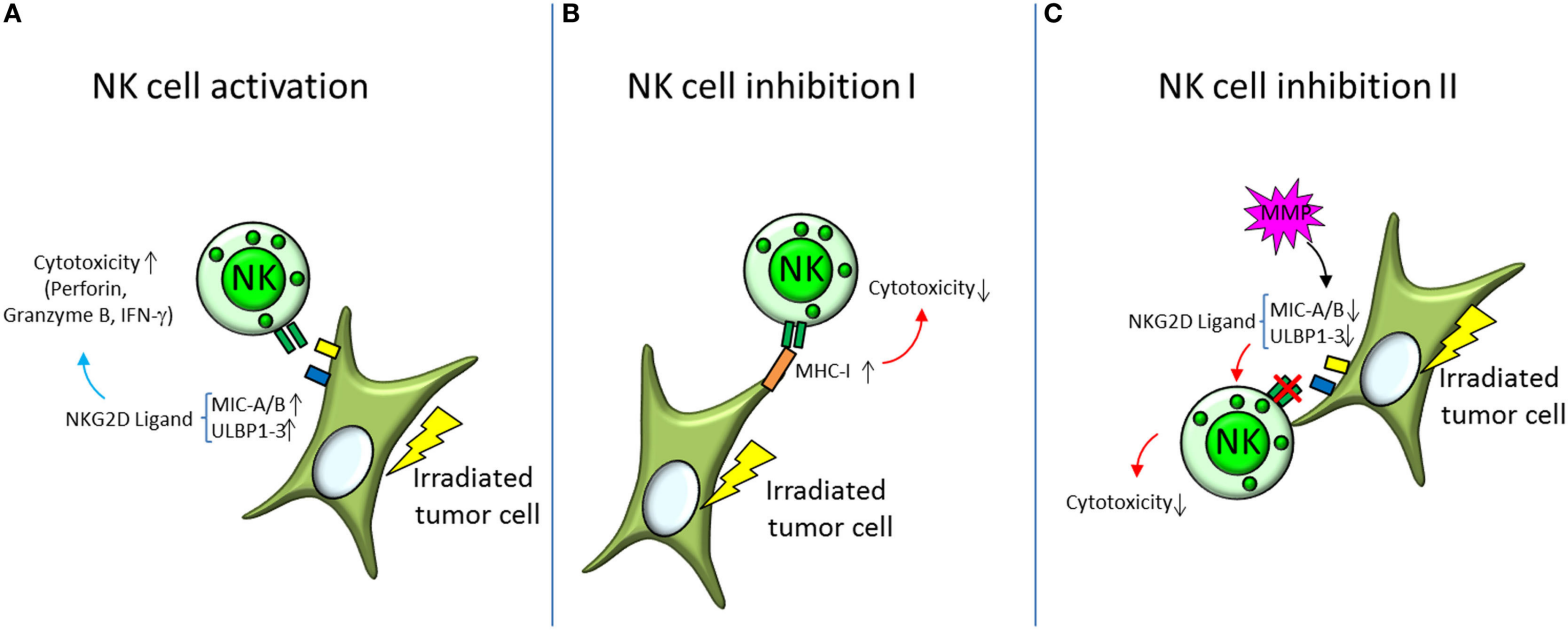
Activation and inhibition of natural killer (NK) cells by irradiated tumor cells. **(A)** Irradiated tumor cells show increased expression of the surface proteins MHC class I chain-related protein A and B (MIC-A/B) and HCMV UL16-binding proteins (ULBP1-3), which are ligands for NK cell activating receptors NKG2D. Activation of NK cells is orchestrated by a balancing of bound activating and inhibiting receptors. Increased expression of NKG2D ligands therefore shifts the balance toward NK cell activation and triggers degranulation of perforine, granzyme B, and interferon γ (IFN-γ)—the NK cells’ mediators of cytolytic activity. **(B,C)** Decreasing NK cell cytotoxicity on the other hand is mediated by different mechanisms. **(B)** Major histocompatibility complex class I (MHC-I) is a ligand for the inhibiting receptors on the NK cell surface and has been found to be elevated in irradiated tumor cells. By increasing the binding of inhibitory receptors, the NK cells’ cytotoxic capabilities are diminished. **(C)** Another mechanism is to decrease the binding to the activating NK cell receptors, like NKG2D. This can be accomplished by cleaving the respective ligands on the target cell surface with matrix metalloproteases (MMP).

The Natural Killer Group 2D [NKG2D, reviewed by Spear et al. (68)] receptor promotes amongst others the activation of NK cells. The human NKG2D receptor recognizes the ligands MHC class I chain-related protein A (MIC-A) and B (MIC-B) and HCMV UL16-binding proteins [ULBP1-6 (68)]. Expression of NKG2D ligands has been found to be increased in irradiated tumor cell lines [NCI-H23, A549 (61, 69)] resulting in enhanced activity of NK cells (summarized in Figure 3) toward tumor cells after X-irradiation (8 Gy). The response was presumed to be triggered by an upregulation of the NKG2D ligands MIC-A/B and ULBP1-3 and could be further increased by inhibition of histone deacetylase (61).

Upregulation of MHC-I molecules and heat-shock proteins may abolish this activation (Figure 3) by induction of an increased expression of inhibitory NK cell surface receptors (61). NK cell activity has also been found to be diminished after cleavage of NKG2D ligands *via* matrix metalloproteases (69, 70).

An enhanced radiotherapy effect mediated by NK cells has been reported after electron irradiation (8 Gy). The cytotoxic effect of NK cells was tested on various cancer stem cell lines (MDA-MB-231, U87MG, A673, and PANC-1) *in vivo*, where mice were inoculated with cultured tumor cells and locally irradiated, then injected with NK cells, and *in vitro*, assessing the NK cytotoxicity directly on irradiated tumor cell (62).

As mentioned above, *in vivo* studies can imply direct irradiation effects on immune cells. A way around this is to inject non-irradiated lymphocytes into the irradiated tumor-bearing host and analyze the effects.

An *in vivo* study explained a reduced tumor volume (RMA-S lymphoma/B16 melanoma) in mice by injected NK cells after 5 Gy total body irradiation. The effect was even more pronounced after prestimulating the NK cells with IL-12, -15, and -18, with highly increased expression of interferon γ (IFN-γ), granzyme B, and perforin. Those prestimulated NK cells were found to have rapidly proliferated in dependence of IL-2 production by CD4+ Th-cells (60).

### Involvement of DCs

Enhanced antitumor response after X-irradiation (PANTAK Therapax DXT 300 Model X-Ray Unit, 42.5 Gy) has been linked to DCs. Intratumoral injection of DC was performed in mice bearing irradiated D5 tumors, resulting in reduced tumor size and increased IFN-γ secretion (71). As shown by Scholch et al. (65), in the *in vivo* (PANC-02 cells in mouse model) antitumor response of immune cells after irradiation (5 Gy × 2 Gy), the CTL mediated response dominates over NK cells, and was shown to be abrogated by depletion of DC, indicating a necessity of DC mediated antigen presentation for the immune cell effectiveness against tumor tissue. Although very promising, the described effects do not take the radiation effect on immune cells into account, since no immune cells were injected after irradiation (65). After X-irradiation (5 Gy × 2 Gy, 3 Gy × 5 Gy, 15 Gy), DC show an increased expression of IL-2R (CD25), which can mediate an increased activation of CD4+ T-cells *via* presentation of the activating IL-2 to the T-cell [and potentially CTL and NK cells as well, although not tested in the study (72)].

### Involvement of Tregs

On the other hand, immunosuppressive Tregs were found to be increased in irradiated tumors in an *in vivo* mouse study, bearing lung and colon tumors (63) as well as in tumors and tumor draining lymph nodes of mice injected with mouse melanoma and lymphoma cell lines (64). The increased presence of Tregs was associated with increased tumor growth and has been hypothesized to depend on Langerhans cells, the DCs in the epidermis (64). Systemic inhibition of Tregs using cycloheximide (CHX) and anti-CD25 antibodies proved to increase the number of CD8+ and CD4+ non-Tregs. Along with those results, CHX and anti-CD25-antibody treatment resulted in enhanced tumor regression, indicating a suppressive function of Tregs (63). In other *in vivo* studies, Tregs were suppressed by blockage of cytotoxic T-lymphocyte associated protein 4 (CTLA4) in mice injected with 4T1 mouse mammary carcinoma or CD-26 murine colon cancer cells. Subsequent radiation exposure with 10 and 12 Gy of γ-irradiation resulted in tumor reduction that was associated with CTL-mediated cytotoxicity (73, 74). In the study by Son et al., the irradiation treatment was augmented with immature DC (74), but due to different irradiation parameters as well as different tumor application of the two studies, the effectiveness of this augmentation cannot be assessed.

### Bystander and Abscopal Effects

Monocytes and T-cells were shown *in vitro* (THP-1 and Jurkat cell lines, respectively) to have increased viability after incubating them with conditioned medium from carbon ion irradiated neuronal tumor cells (SH-SY5Y and U87; Carbon ions 165 MeV/n, LET 30 keV/µm, 1–5 Gy), as well as decreased migration of THP-1, hinting at more in-depth interactions of immune cells in response to radiation (75).

Radiation therapy with an electron beam (fractionated 8 Gy on three consecutive days; Varian Truebeam linear accelerator) has been shown to slow tumor growth of mice bearing 67NR tumors *in vivo* in an abscopal manner (76). In the same model, after enrichment of DC using DC growth factor Flt3-L (Fms-related tyrosine kinase-3 ligand), abscopal tumor size reduction was observed after low doses (2–6 Gy) of γ-irradiation (^60^Co source). The effect was proven to be T-cell dependent, as abscopal tumor size was not influenced in T-cell deficient mice (54).

The systemic inhibition of Tregs using CHX and anti-CD25-antibodies in an *in vivo* tumor-bearing mouse model (lung and colon carcinoma) or *via* CTLA4 blockage in an *in vivo* tumor-bearing mouse model (colon carcinoma) after irradiation of the tumor resulted in reduced growth of distant non-irradiated tumor cells (63, 74). The indicated suppressive action of Tregs on antitumor responses can thereby also be expected non-irradiated tumors.

These studies show that irradiation of tumor cells or tissue has long-ranging effects on different immune cell subpopulations. This results in activation of CTL and NK cells, supported by increased activity of DCs, which meets an orchestrated immune suppressive response initiated by Tregs. Activation of CTL and NK cells was shown in *in vitro* and *in vivo* studies, Treg activation only *in vivo*. As a broad variety of neoplastic cell lines activated these immune cell populations, the tumor cell type seems to have no apparent influence on immune cell activation.

## Cytokines and Chemokines

### The Tumor Milieu

The presence of immunosuppressing cyto- and chemokines is vital to the development and progression of tumor cells. The tumor cells themselves can secrete factors that protect them from lysis *via* CTLs or NK cells or elicit cytokine expression in other cells that enable tumor survival, most notably are TGF-β and IL-10.

Transforming growth factor β has been shown to reduce a wide variety of antitumor immune functions. It inhibits growth of immune cells and reduces IL-2, IL-2R, IFN-γ, and NKG2D expression resulting in impairment of their activity. Furthermore, downregulation of MHC-I molecules on the tumor cell surface reduces their susceptibility to CTL-mediated tumor cell lysis. Expression of TGF-β by several tumor types has been reported (77, 78).

Interleukin 10 is one of the immune system’s “Off-Switches,” known for its regulatory characteristics in suppressing inflammatory responses (79). It effectively reduces antigen-presentation, Th1 responses, NK cell cytokine expression, and functions of monocytes and macrophages. An important way of inactivating the inflammatory immune response is by reducing the abilities of DCs to present antigens and to produce proinflammatory cytokines such as IL-12 (80). IL-12 can promote NK-mediated actions against tumor tissue. Among other factors associated with tumor growth are TNF, IL-1, IL-6, IL-8 [C-X-C motif chemokine ligand 8 (CXCL8)], IL-11, IL-17a, IL-22, acute phase proteins, CCL20, PGE2, colony stimulating factor-1 (CSF-1)/macrophage CSF (M-CSF), vascular endothelial growth factor (VEGF), and granulocyte-macrophage CSF (GM-CSF) (81–86).

The field of cytokines promoting tumor development and progression is vast and has been reviewed elsewhere (81–85). The communicative relationship of cytokines in radiation biology, as well as general notions on their functions, has been extensively reviewed by Schaue et al. (87). One can suspect that modulation of cytokine expression may be able to accomplish long ranging effects in terms of non-targeted responses after irradiation exposure. In this review, the focus lies on the modulation of cytokine expression after exposure to ionizing radiation from differing radiation qualities and irradiation schemes.

### Impact of Irradiation of Tumor Cells on Cytokine Expression

**Figure 4:**
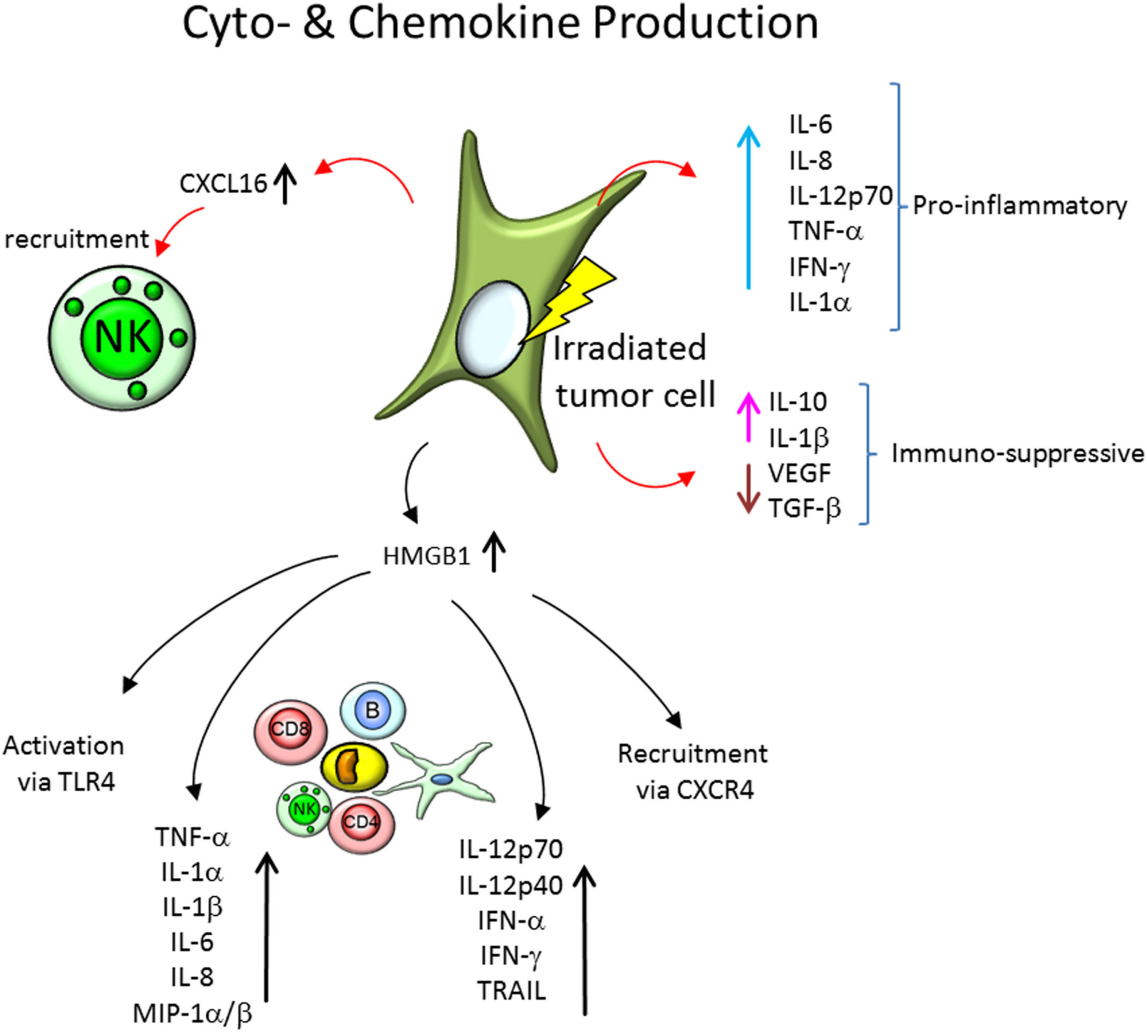
Cytokine and chemokine expression by irradiated tumor cells, recruitment of immune cells and cytokine expression of the involved immune cells. Tumor cells express a plethora of soluble factors, cytokines and chemokines, and after irradiation, the secretion profile is modified. On the one hand, proinflammatory cytokines, like interleukin-6 (IL-6), IL-8, IL-12p70, tumor necrosis factor α (TNF-α), interferon γ (IFN-γ), and IL-1α, are increasingly expressed in tumor cells models *in vitro* and *in vivo*. On the other hand, the expression of immune-suppressive soluble factors is modified. IL-10 and IL-1β expression is increased, but secretion of vascular endothelial growth factor (VEGF) and transforming growth factor-β (TGF-β) is reduced. Further, chemokines, like CXCL16, are increasingly expressed and initiate recruitment of natural killer (NK) cells and other immune cells. The secretion of the damage-associated molecular pattern molecule high mobility group box 1 (HMGB1) is elevated as well in irradiated tumor cells, which leads to a activation of immune cells *via* the toll-like receptor 4 (TLR4), recruitment of immune cells *via* chemokine receptor CXCR4, as well as modification of cytokine expression of peripheral blood mononuclear cells.

Since the tumor environment is of immunosuppressive nature, the question arises, how irradiation of tumor cells modulates the cytokine responses that induce or further suppress the immune response. The cytokine and chemokine response of diverse tumor cell lines is shown in Table 2 and Figure 4.

**Table 2:** Cyto- and chemokine response and damage-associated molecular patterns (DAMPs) release by tumor cells after irradiation.

Tumor cell	Radiation quality	Dose	Study type	Cytokine/chemokine	Expression	Reference
4T1, 67NR, HTB-20 (breast carcinoma)	γ-Irradiation (^137^Cs source)	2–12 Gy	*In vivo*	CXCL16	⇑	(88)
			*In vitro*			

T98G (glioblastoma)	γ-Irradiation (^60^Co source)	1 Gy	*In vitro*	IL-6, IL-8	⇑	(89)

4T1, 67NR (breast carcinoma), B16/F10 (melanoma), MC57 (fibrosarcoma), MCA38 (colon carcinoma)	γ-Irradiation (^137^Cs source)	12 Gy	*In vivo*	CXCL16	⇑	(90)

A549, TE2, KYSE70 (esophageal squamous), NCI-H460 (large cell carcinoma), WiDr (colon adenocarcinoma), MCF-7, NCI-H1703 (lung), DU-145, PC-3 (prostate), HCT-15 (colorectal), SW480, T98G and U251MG	Photonic	2.1–15 Gy	*In vitro*	HMGB1	⇑	(91–95)

DF-19, BW-225 (squamous cell carcinoma)	Ionizing radiation (not specified)	2 Gy	*In vitro*	CXCL1, CXCL12	=	(96)

HT1080 (colorectal tumor), U373MG, HT29, A549, MCF-7	γ-Irradiation (^60^Co source)	2 Gy, 6 Gy, 3 Gy × 2 Gy	*In vitro*	Flt3-L, G-CSF, GM, CSF, IL-1β, IL-6, IL-8, IL-15, IP-10, MCP-1, TNF-α, TGF-β, VEGF	⇑	(97)
				G-CSF, GM-CSF, IL-1β, IL-6, IL-8, MCP-1, TNF-α, TGF-β	⇓	

SW480 (colorectal)	X-rays	5 Gy × 2 Gy, 3 Gy × 5 Gy, 15 Gy	*In vitro*	IL-6, IL-8, IL-12p70, TNF-α, IL-10, IL-1β	⇑	(72)

LN-229 (glioma)	γ-Irradiation (Nordion GC40 Gammacell irradiator)	10–30 Gy	*In vitro*	IL-6	⇑	(98)
				IL-8, CXCL1 (only mRNA)		

NCI-H446 (lung)	γ-Irradiation (^137^Cs source)	8 Gy	*In vitro*	TNF-α, IL-1α	⇑	(99)
	Carbon ions (290 MeV/n, LET 13 keV/µm)	2 Gy		TNF-α	⇑	

RipTag5 mice (spontaneous insulinoma)	γ-Irradiation (^60^Co source)	2 Gy	*In vivo*	TNF-α, IL-12p70, IFN-γ	⇑	(100)
				VEGF, TGF-β	⇓	

MCF7, SKBR3, and MDA-MB231 (breast)	γ-Irradiation (^137^Cs source)	10–20 Gy	*In vitro*	CXCL16	⇑	(101)

NR-S1 and SCCVII (squamous cell carcinoma), NFSa, #8520 (fibrosarcoma)	γ-Irradiation (^137^Cs source)	30–50 Gy	*In vivo*	CCL3 (only mRNA)	⇑	(102)
	Carbon ions (290 MeV/n, LET 50 keV/µm)	30 Gy		CCL3, CXCL2 (only mRNA)		

TE2, KYSE70, A549, NCI-H460 and WiDr	Carbon ions (290 MeV/n, LET 30 keV/µm)	0.9–3.5 Gy (iso-survival dose D_10_^a^)	*In vitro*	HMGB1	⇑	(93)

*⇑ up, ⇓ down*.

*^a^The D_10_ dose represents the radiation dose required to reduce the surviving fraction to 10%*.

Fractionated irradiation (5 Gy × 2 Gy, 3 Gy × 5 Gy, 15 Gy) of human colorectal adenocarcinoma cells (SW480 cell line) with X-rays has been reported to increase expression and secretion of proinflammatory cytokines IL-6, IL-8, IL-12p70, and TNF-α by DCs. The immunosuppressive cytokine IL-10 and the proinflammatory cytokine IL-1β were insignificantly increased without impeding antitumor response of Th1-cells (72).

The glioblastoma cell line T98G expressed and secreted an elevated level of IL-6 and IL-8 after γ-irradiation (1 Gy) (89). In addition to IL-6 and IL-8, CXCL1 expression was induced by high γ-radiation doses (10–30 Gy) in LN-229 glioma cells, this was observed for several days after irradiation (98).

Desai et al. (97) analyzed cytokine expression in the tumor cell lines HT1080, U373MG, HT29, A549, and MCF-7, using a single dose (2 and 6 Gy) and fractionated doses (2 Gy × 3 Gy) of γ-rays. Amongst the cytokines tested were TNF-α, IL-1β, IL-6, TGF-β, monocyte chemotactic protein 1 (MCP-1/CCL2), IL-15, VEGF, G-CSF, GM-CSF, Flt3-L, and IFN-γ-induced protein 10 (IP-10). While some cytokines (TNF-α, IL-8, IL-15, GM-CSF, and TGF-β) were highly upregulated after 6 Gy single-dose γ-irradiation, the expression profile strongly depended on the dose (TGF-β was downregulated in HT1080 cells at 2 Gy, highly upregulated at 6 Gy, and moderately upregulated in the fractionated irradiation scheme), cell line (downregulation of IL-6 in every irradiation scheme of U373MG cells but upregulation in HT1080 and A549 cells) and fractionation (IL-1β was downregulated in HT29 cells at 2 and 6 Gy single-dose irradiation but upregulated in the fractionated irradiation scheme).

In a mouse tumor model (RipTag-5 transgenic mice), TNF-α, IL-12p70, and INF-γ expression was found to be elevated, while VEGF and TGF-β were decreased after irradiation with 2 Gy γ-rays (100).

Tumor necrosis factor α and IL-1α were also reported to be released in H446 lung cancer cells after irradiation with γ-rays (8 Gy), but only TNF-α after irradiation with accelerated carbon ions (290 MeV/n, LET 13 keV/µm, 2 Gy) (99). IL-1 can act as stromal growth factor in tumors (103).

In conclusion, exposure to X-rays or γ-rays in therapeutic dose ranges (in fractionated or single-dose regime) modulates the expression of cytokines in many different tumor cell lines and also spontaneous tumor models.

### Impact of Irradiation of Immune Cells on Cytokine Expression

Monocytes (THP-1 cell line) expressed reduced levels of the activating factors IL-15, IL-17, macrophage inflammatory protein 1β (MIP-1β, also known as CCL3) and IL-2 as well as increased levels of Treg-attracting IP-10 [CXCL10 (104)], Rantes (CCL5) and immunosuppressive VEGF (105) 24 h after irradiation with 1.5 Gy α-particles (^241^Am Source, LET 127 keV/µm) (106). Irradiation of THP-1 derived macrophages with 0.5–20 Gy carbon ions (18.3 MeV/n, LET 108 keV/µm) has been shown to result in decreased TNF-α and IL-6 expression. Only extremely high doses (50 Gy) of carbon ions resulted in this study in an increased IL-6 expression (107). Irradiation of monocytes and macrophages with α-particles or accelerated carbon ions in therapeutic dose ranges (fractionated scheme) may therefore negatively modulate the immune response against tumor cells.

### Release of DAMPs

Damage-associated molecular patterns are secreted or released biomolecules that can initiate inflammatory responses upon binding to recipient receptors. Among those biomolecules are DNA molecules that are recognized by PRR anywhere outside the cell nucleus, or damaged RNA, which may be released in response to damages induced by ionizing radiation. Certain signaling proteins may also be recognized by PRR and stimulate immune functions.

One of those proteins is the high mobility group box 1 (HMGB1), a protein that under normal conditions binds to chromosomal DNA and facilitates nucleosomal structure maintenance and regulates gene expression. Acting as a DAMP (Figure 4), HMGB1 can support recruitment of immune cells *via* the chemokine receptor CXCR4 (which is bound by CXCL12) and activate immune responses *via* toll-like receptor 4 (TLR4) or induce caspase-1-dependent apoptosis (93, 95, 108) as well as DC maturation, Th1 polarization (109), and IFN-γ release of NK cells (110). It has been shown to be released after 2–15 Gy, X- and 0.9–3.5 Gy carbon ion irradiation by normal human fibroblasts (GM0639) and human bronchial epithelial cells (16HBE), as well as by the tumor cell lines A549, TE2, KYSE70, NCI-H460, WiDr, and the mouse melanoma cell line B16-F10 (93–95). Similar findings were reported in tumor cell lines of various tissue origins (MCF-7, NCI-H1703, DU-145, PC-3, HCT-15, SW480, T98G, and U251MG cells) (91, 92). Upon TLR9 stimulation, HMGB1 induces expression of IL-12p70, IL-12p40, IFN-α, IFN-γ, and TRAIL in DCs (111).

HMGB1 has also been indicated to induce NF-κB activity, as measured by p65 translocation as well as IκBα degradation, in presence of PRR CD14 and TLR4 (112). HMGB1 also induces increased TNF-α expression in human peripheral blood mononuclear cells, as well as TNF-α, IL-1α, IL-1β, IL-1RA, IL-6, IL-8, MIP-1α, and MIP-1β in human monocytes, but not IL-10 or IL-12 (113).

This indicates that inflammatory protein expression of immune cells may be in part due to stimulation *via* HMGB1 acting as DAMP after irradiation injury. As part of the bystander response of immune cells, this HMGB1 induced expression of cytokines may lead to prompting further immune action such as CD8+ T-cell or NK cell mediated killing of irradiated tumor tissue. There is very little data available regarding HMGB1 modulation by proton or carbon ion irradiation. This calls for more research in this upcoming and promising radiotherapy approach—especially in context of DAMP interaction with immune cells. While the primary signaling pathways for interactions of HMGB1 with any leukocyte population can be elucidated using X-irradiation, the effect of particle irradiation on this intercellular communication can only be assessed with specifically designed experiments for this question.

### Bystander Cytokine Expression

Besides the cyto- and chemokine expression of irradiated tumor or immune cells, bystander cells not directly hit by radiation might modify their gene expression profile. In coculture, U937 macrophages have been shown to secrete TNF-α and IL-1α (IL-1α not at high doses) after irradiation of NCI-H446 lung cancer cells with γ-rays (^137^Cs Source, 8 Gy) but only TNF-α after irradiation with accelerated carbon ions (290 MeV/n, LET 13 keV/µm, 2 Gy) (99). Microbeam irradiation of 0.45% of a THP-1 derived macrophage population with 5 Gy carbon ions (18.3 MeV/n, LET 108 keV/µm) using a heavy ion microbeam resulted in significantly reduced expression of TNF-α and IL-6 (107).

### Chemokines and Lymphocyte Recruitment

The release of chemokines by irradiated cells and build-up of a chemokine gradient results in recruitment of selected immune cell populations to the irradiation site.

The chemokine CX3CL1 can recruit osteoclasts which are formed by fusion and differentiation of monocytes (114). This might have clinical relevance for osteolytic tumors.

Expression of CXCL16, the only known ligand for chemokine receptor CXCR6—expressed on NK cells, is increased in various breast cancer (MCF7, SKBR3, and MDA-MB231, 4T1, 67NR, HTB-20), melanoma (B16/F10), fibrosarcoma (MC57), and colon carcinoma (MCA38) cell lines after γ-irradiation (2–20 Gy) (88, 90, 101). This increased expression of CXCL16 facilitates an enhanced migration NK cells (Figure 4) toward the tested tumor cells (101).

Macrophage inflammatory protein 1α (CCL3) is a T-lymphocyte/monocyte derived chemokine recruiting CCR1/CCR5 expressing leukocytes (monocytes, DC, NK-, and T-cells). Administration of an active CCL3 agent (ECI301) resulted in reduction of tumors *in vivo* (Colon26 adenocarcinoma, MethA fibrosarcoma and Lewis lung carcinoma cells in mice) after electron irradiation (6 MeV electron beam, 6 Gy). Depletion of CD8+ T-cells reduced the antitumor effect of CCL3 administration indicating radiation induced recruitment of this cell population to the tumor site (115).

### Abscopal Effects

CCL3 administration also served to reduce tumor size of non-irradiated tumors in the *in vivo* model used by Shiraishi et al. (115). This effect was dependent CD4+ Th cells and NK cells, as depletion of those cell populations has shown. This indicates a CCL3 dependent recruitment of those populations to the non-irradiated tumor after irradiation (115).

## Conclusion

After the initial irradiation of tumor cells, the RIBE can contribute to a more effective elimination of the tumor by recruiting immune cells to the tumor and by activating immune cells at the tumor site. The interactions between irradiated cancer as well as irradiated or bystander and abscopal immune cells are manifold. These radiation-induced interactions of immune cells in the tumor response are being elucidated for photonic radiation, but the effects of protons and carbon ions are largely unknown. First studies indicate a trend toward stronger cytokine expression by the tumor cells after carbon ion exposure. Extensive research is still necessary to unravel the mechanisms of the interplay of immune cells with the irradiated tumor in order to promote a more efficient therapy. The dependence on radiation quality, irradiation scheme as well as tumor origin makes a unifying statement about the expression of cytokines by tumor cells incredibly difficult. The ability of cytokines—as well as danger signals like HMGB1—to shift the immunoevasive tumor toward a state of damaged tissue engages the whole immune machinery to intervene against the neoplasia.

A cancer therapy approach using ionizing radiation and immune modulation has reached the clinical study status (76). Especially modulations that use agents promoting either activation or recruitment of immune cells are being considered. Postirradiation injection of non-irradiated endogenous immune cells, such as CTL, NK cells, and DC, to clear up the irradiated tumor more effectively are worth further investigation.

The use of radiation qualities that can more precisely target tumor cells, such as protons and carbon ions, in combination with immune therapy seems like a promising approach toward even more efficient cancer treatment, as the immune promoting effects of ionizing radiation can be supported by the local tumor control.

## Author Contributions

CEH had the idea for this review, designed it and wrote the abstract and the introduction, designed Figure 1, contributed the immune cells for Figures 2–4 and redesigned Figures 2–4, inserted the references, corrected, and edited all other parts. SD wrote all chapters following the introduction, drafted Tables 1 and 2, and invented Figures 2–4.

## Conflict of Interest Statement

The authors declare that the research was conducted in the absence of any commercial or financial relationships that could be construed as a potential conflict of interest.

## Abbreviations

APCs, antigen-presenting cells; CCL, C–C motif chemokine ligand; CCR, C–C chemokine receptor type; CD, cluster of differentiation; Cdkn1a, cyclin dependent kinase inhibitor 1A; CEA, carcinoembryonic antigen; CHX, cycloheximide; CSF-1, colony stimulating factor-1/M-CSF, macrophage CSF/G-CSF, granulocyte CSF/GM-CSF, granulocyte-macrophage CSF; CTL, cytotoxic T-cell; CTLA4, cytotoxic T-lymphocyte associated protein 4; CXCL, C–X–C motif chemokine ligand; CXCR, C–X–C chemokine receptor type; DAMPs, damage-associated molecular patterns; DCs, dendritic cells; Flt3-L, Fms-related tyrosine kinase-3 ligand; HLA, human leukocyte antigen; HMGB1, high mobility group box 1; HPC, hematopoietic progenitor cell; HSC, hematopoietic stem cell; ICAM-1, intercellular adhesion molecule 1; IFN-γ, interferon γ; IL, interleukin; IP-10 (CXCL10), IFN γ-induced protein 10; LET, linear energy transfer; MCP-1 (CCL2), monocyte chemotactic protein 1; MeV/n, Megaelectronvolt per nucleon; MHC-I and II, major histocompatibility complex class 1 and 2; MIC, MHC class I chain-related protein; MIP-1β (CCL3), macrophage inflammatory protein 1β; MUC-1, mucin-1; NK, natural killer; NF-κB, nuclear factor κB; NKG2D, Natural Killer Group 2D; PGE2, prostaglandin E2; PRRs, pattern recognition receptors; RIBEs, radiation-induced bystander effects; RNS, reactive nitrogen species; ROS, reactive oxygen species; TGF-β, transforming growth factor β; Th, T helper; TLR, toll-like receptor; TNF-α, tumor necrosis factor α; Tregs, regulatory T-cells; ULBP, UL16-binding proteins; VEGF, vascular endothelial growth factor.
